# Clinical features and ultrasound findings of a rare musculoskeletal system disease–neuromuscular choristoma

**DOI:** 10.1186/s12891-022-05238-4

**Published:** 2022-05-17

**Authors:** Wen Guo, Hong Wang, Tao Chen, Wei Yang, Shu-Feng Wang, Shan-Lin Chen

**Affiliations:** 1 Department of Ultrasound, Beijing Jishuitan Hospital, Fourth Clinical College of Peking University, No.31 Xinjiekou East Street, Xicheng District, Beijing, 100035 China; 2 Key Laboratory of Carcinogenesis and Translational Research (Ministry of Education/Beijing), Department of Ultrasound, Peking University Cancer Hospital and Institute, Haidian District, 52 Fucheng Road, Beijing, 100142 China; 3 Department of Hand Surgery, Beijing Jishuitan Hospital, Fourth Clinical College of Peking University, No.31 Xinjiekou East Street, Xicheng District, Beijing, 100035 China

**Keywords:** Neuromuscular choristoma, Ultrasound, Fibromatosis, Desmoid-type fibromatosis

## Abstract

**Background:**

Neuromuscular choristomas (NMCs), are extremely rare developmental lesions that, have been previously established associated with recurrent fibromatosis after surgery, leading to several operations or even amputation. However, reports on the ultrasound imaging features and clinical conditions of NMCs are rare. The purpose of this study is to describe the ultrasound features and clinical analysis of NMCs to provide suggestions to identify the optimal management strategy.

**Methods:**

From September 2020 to September 2021, 7 patients with a confirmed diagnosis of NMC who underwent ultrasound examination in our department were enrolled in our study. Physical examinations were performed to detect motor deficits, sensory deficits, neuropathic pain, limb undergrowth, muscular atrophy, cavus foot and bone dysplasia. Ultrasound imaging was performed and investigated both in affected nerves and neuromuscular choristomas associated desmoid-type fibromatosis (NMC-DTF). All patients had a definite history and regular follow-up. The clinical course, physical examinations, ultrasound features and pathologic results of NMC patients were analyzed.

**Results:**

Seven patients with an average age of 7.0 ± 7.2 years (range: 2–22 years) were enrolled in our study. The affected nerves included the sciatic nerve (6 cases) and the brachial plexus (1 case). Six patients (85.7%) presented with limb undergrowth, 6 (85.7%) with muscular atrophy, and 5 (71.4%) with cavus foot deformity. Based on ultrasound findings, all the visibly affected nerve segments presented with hypoechoic and fusiform enlargement with intraneural skeletal muscle elements. Five patients (71.4%) had NMC-DTFs at the site of the affected nerve. All NMC-DTFs were shown as hypoechoic solid lesions adjacent to the nerve and were well circumscribed. In the subset of the surgery group, all 5 patients presented with progression to NMC-DTFs at the site of the NMCs. No fibromatosis was detected in the other two nonsurgical patients.

**Conclusions:**

Understanding the typical ultrasound features and clinically associated conditions would support the early diagnosis of this rare disease. When a potential diagnosis is determined, an invasive procedure such as biopsy or resection might not be a good choice given the frequent occurrence of complications such as aggressive recurrence.

## Background

Neuromuscular choristomas (NMCs), previously known as benign triton tumors, are rare congenital lesions with differentiated mature skeletal muscle tissue found within peripheral nerve fascicles [1–5]. Patients often present with NMCs during childhood, adolescence, but occasional during adulthood [1, 6]. NMCs commonly affect the sciatic nerve (the most common location of a NMC), or the brachial plexus but are known to involve other sites, such as the trigeminal nerve [7], oculomotor nerve [8, 9], or the internal auditory meatus [10]. Histologically, NMC is characterized by the presence of mature muscle fibers inside the endoneurium intercalated among nerve fibers[11]. In most of the reported cases [6, 12–14], the lesion was solitary, associated with a major nerve, and composed of disorganized mature skeletal muscle fibers admixed with nerve twigs. Desmoid-type fibromatosis(DTF) at the NMC site may occur and seems to be incited by surgical biopsy [15, 16]. Aggressive neuromuscular choristomas associated desmoid-type fibromatosis (NMC-DTF) often require multiple operations, radiation and chemotherapy, or even amputation [17]. Therefore, it is important to identify the clinical and imaging features of NMCs to avoid invasive procedures such as biopsy or resection and prevent aggressive fibromatosis. The purpose of this study was to describe the clinical and ultrasound features of NMCs and to determine the optimal management strategy.

## Methods

This prospective study was approved by the Institutional Review Board (approval number 201805–09). All methods were performed in accordance with relevant guidelines and regulations. Written informed consent was obtained from all patients/legally authorized representatives of under 16 age participants.

### Patients

After encountering a case of DTF found to have an occult NMC, we performed a prospective study to confirm a new diagnose of underlying NMC. From September 2020 to September 2021, seven patients with a diagnosis of NMC and a definite history were enrolled in our study. The inclusion criteria were as follows: (1) pathology-proven or clinic-radiological NMC diagnosis; (2) fusiform enlargement of any nerve; and (3) intraneural soft-tissue components with echo intensity comparable to surrounding skeletal muscle.

The exclusion criteria for this study included: (1) enlargement of nerves caused by trauma or surgery; (2) other peripheral nerve tumors such as neurofibroma, schwannoma, perineurioma and lipomatosis; and (3) missed follow-up.

### Clinical and physical examination

The clinical and physical examinations were collected, including age, sex, NMC location, the presence of motor deficit, sensory deficit, neuropathic pain, limb undergrowth, muscular atrophy, cavus foot, and bone dysplasia.

### Ultrasonography

All enrolled patients received ultrasound examination to evaluate the disease features and progression at each follow-up. All sonographic examinations were performed by one of two radiologists (W.G., T.C.) with more than 10 years of experience in musculoskeletal ultrasound procedures. The ultrasound features that were evaluated included NMC location, morphology and the internal characteristics of the affected nerves, presence of NMC-DTF, tumor size, margin, echo intensity, color Doppler blood flow grade, spatial relationship between NMC-DTF and the affected nerves and local recurrence and/or progression.

Because it was not the first-time for some patients (especially for recurrent NMC-DTFs) to ask for medical consultations and/or treatment in our institution, we collected information on past history including operation and postoperative course, pathological results, treatment process, recurrence frequency, and neurologic motor and sensory examination at each follow-up as well. All the clinical courses, physical examinations, ultrasound features and available pathologic results of NMC patients were reviewed and evaluated carefully.

## Results

There were 4 males and 3 females, with an average age of 6.3 ± 8.0 years (range: 1–22 years of age) enrolled in our study. Among the seven patients, five patients were diagnosed based on a nerve biopsy or/and surgery; two patients were diagnosed due to the presence of characteristic clinical and imaging features of an NMC; therefore, they did not undergo a nerve biopsy based upon the current “no touch” principle. The sciatic nerve (6 cases, 85.7%) and the brachial plexus (1 case, 14.3%) were the only two affected nerves.

### Clinical presentation and physical examination

**Fig. 1:**
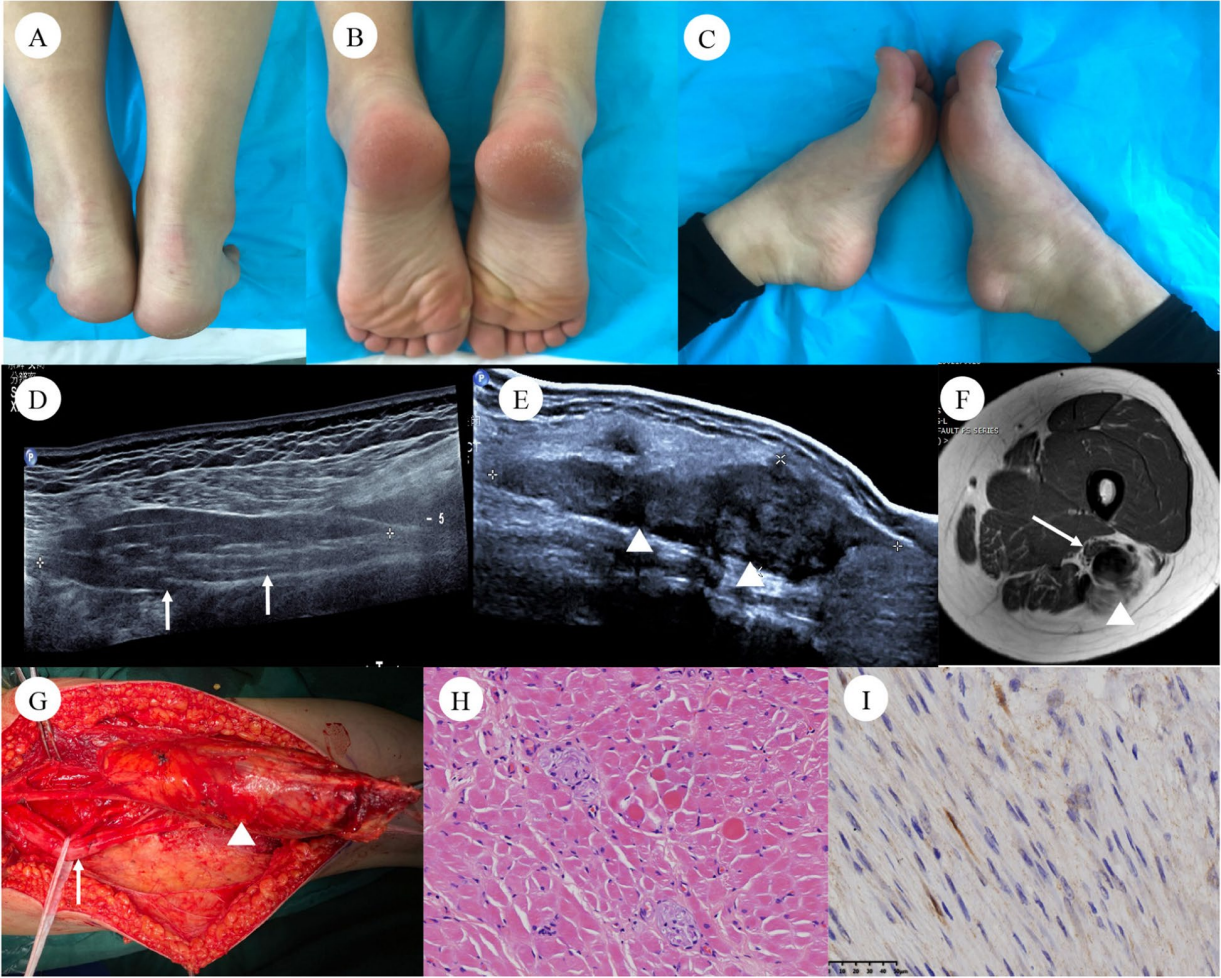
Images of patient #1. A 22-year-old female presented with left limb undergrowth (**A, B**), a left cavus foot was shorter than the right foot (**C**). Ultrasound images demonstrated hypoechoic and fusiform enlargement of the left sciatic nerve (diameter: 2.1 cm). The echo intensity of the intraneural soft-tissue elements was similar with the surrounding skeletal muscle (**D**). An irregular hypoechoic solid lesion was intimately associated with the distally affected sciatic nerve (12.6 × 3.3 cm) (**E**). MRI demonstrated an abnormal thickening of the sciatic nerve (↑) and a solid mass with irregular borders (△) (**F**). The patient underwent surgical tumor resection; note that the left sciatic nerve revealed marked thickening (↑) with desmoid-type fibromatosis (DTF) in branches of the nerve (△) (**G**). A cross-section micrograph (**H & E**) of the affected sciatic nerve showed endoneurial intercalation of mature skeletal muscle fibers among the peripheral nerve fascicles and diagnosed neuromuscular choristomas (NMC) (**H**). Beta-catenin immunohistochemical staining shows both aberrant nuclear staining and cytoplasmic staining in the DTF (**I**)

**Fig. 2:**
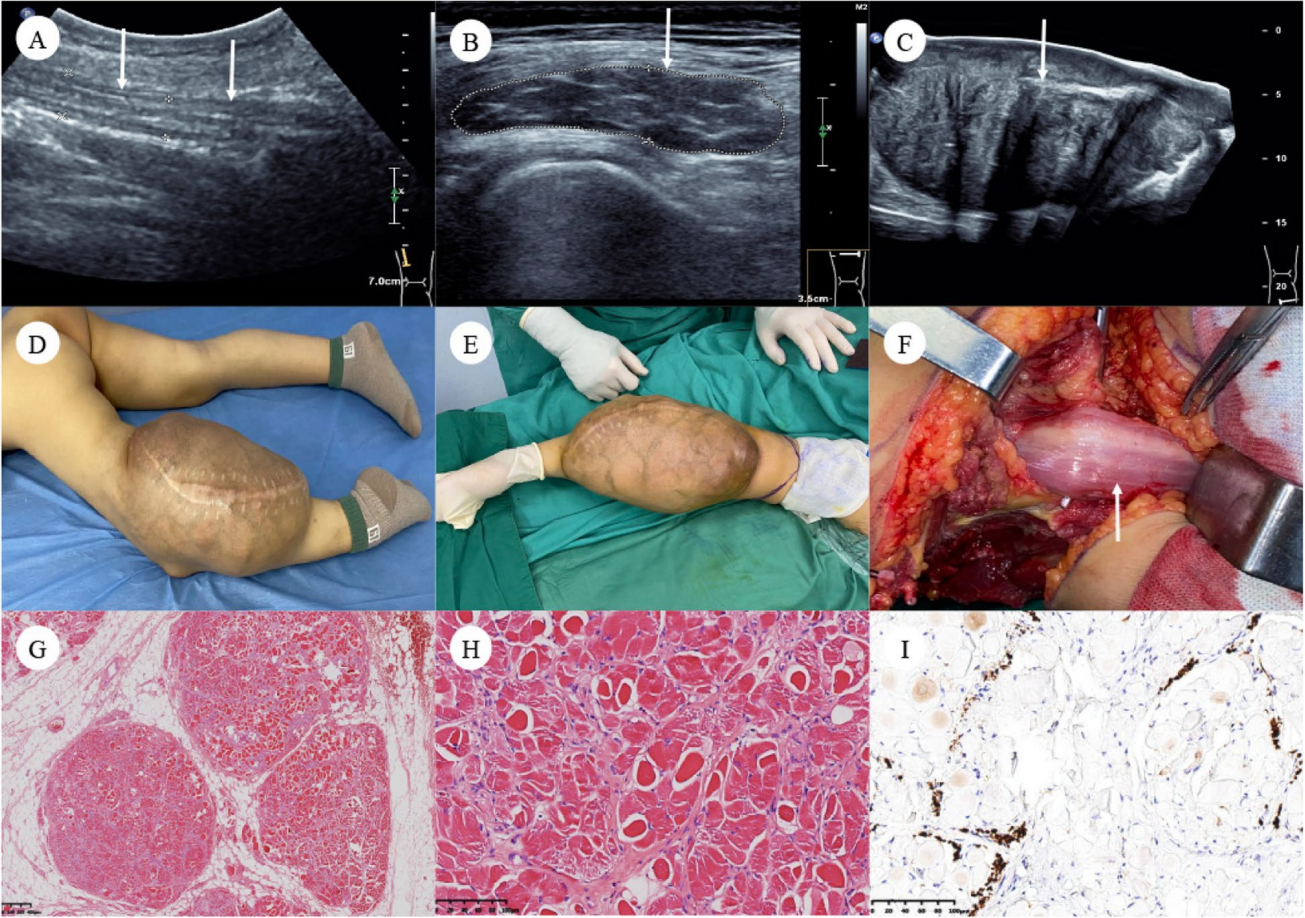
Images of patient #3. A 6-year-old boy with a history of multiple recurrent desmoid-type fibromatosis (DTF) after surgery. Ultrasound images showed hypoechoic and fusiform enlargement of the left sciatic nerve in the middle of the thigh in both longitudinal sections (diameter: 1.2 cm) (**A**) and cross-sections (cross-sectional area 2.2cm^2^) (**B**). DTF was shown as an irregular solid lesion with heterogeneous internal echo at the side of the affected nerve (24 × 17 × 12 cm) (**C**). Recurrent DTF developed as a giant mass of the left lower limb (**D**, **E**). After two rounds of chemotherapy and six rounds of high-intensity focused ultrasound knife (HIFU) treatment, the patient underwent amputation surgery; note that the left sciatic nerve was markedly thickened with skeletal muscle tissue within nerve (**F**). Micrograph (H&E) of the cross-section of the affected sciatic nerve showed endoneurial intercalation of mature skeletal muscle fibers among the peripheral nerve fascicles and diagnosed neuromuscular choristomas (NMC) (**G**, **H**). NMC-DTF showed strong beta-catenin expression in immunohistochemical staining (**I**), and a CTNNB1 p. S45F mutation was identified

**Fig. 3:**
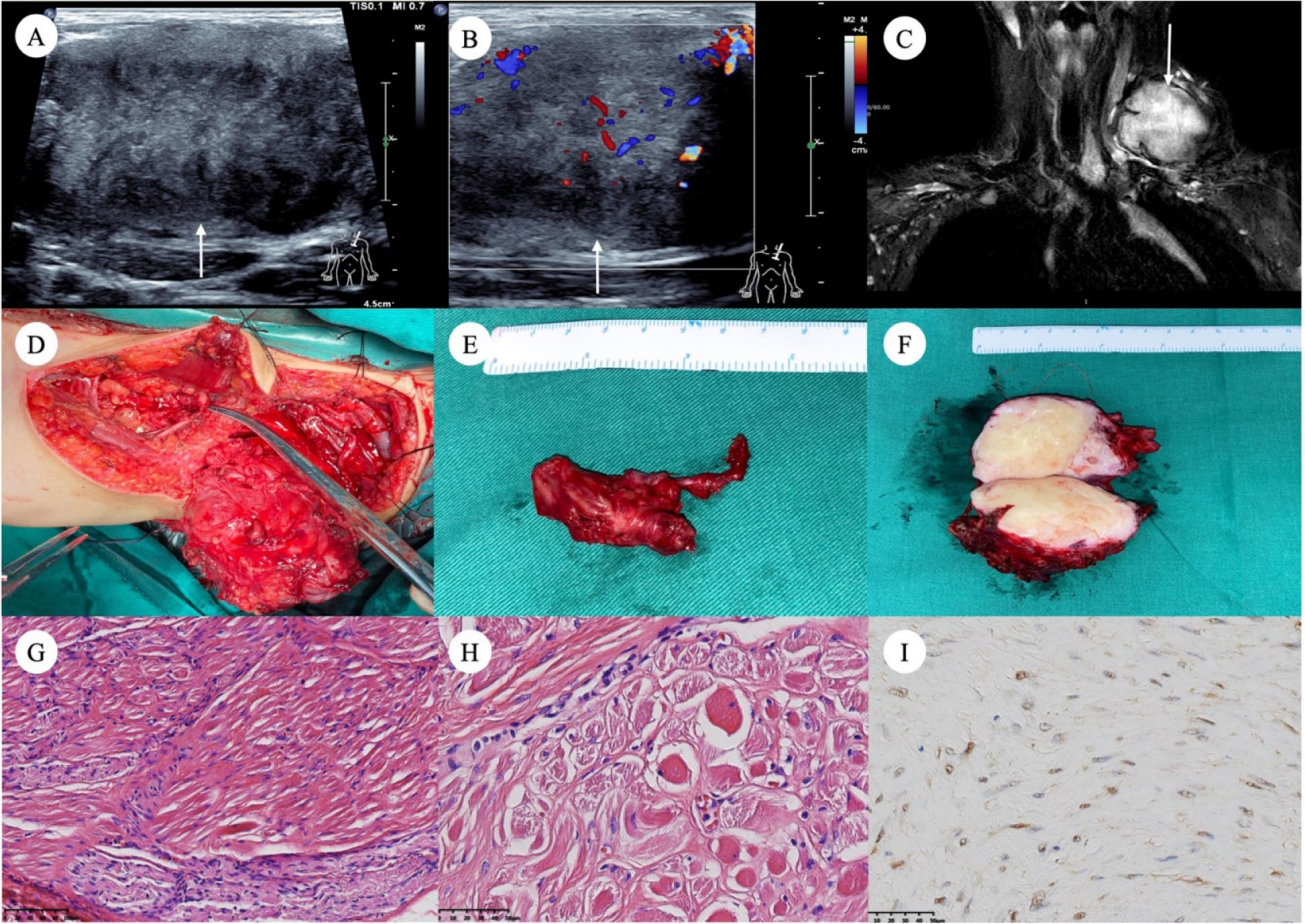
Images of patient #4. A 10-year-old girl with a left cervical mass and neuropathic pain. Ultrasound images showed an irregular heterogeneous hypoechoic solid lesion at the course of the left brachial plexus (**A**) with color Doppler flow imaging grade I (**B**). MRI demonstrated a 5.5 × 5.5 × 3.3 cm mass with an irregular shape consistent with ultrasound images and showed that the tumor invaded part of the left brachial plexus (**C**). The patient underwent tumor and affected brachial plexus resection and nerve transplantation; note that the left brachial plexus was partly surrounded by the tumor, and partly pressed and narrow (**D**). Surgical specimens showed thickened C7 nerve root (**E**) and desmoid-type fibromatosis (DTF) (**F**). Micrograph (H&E) of the cross-section affected brachial plexus showed endoneurial intercalation of mature skeletal muscle fibers among the peripheral nerve fascicles and diagnosed neuromuscular choristomas (NMC) (**G**, **H**). Immunohistochemical staining demonstrated strong beta-catenin expression in NMC-DTF cells (**I**)

**Fig. 4:**
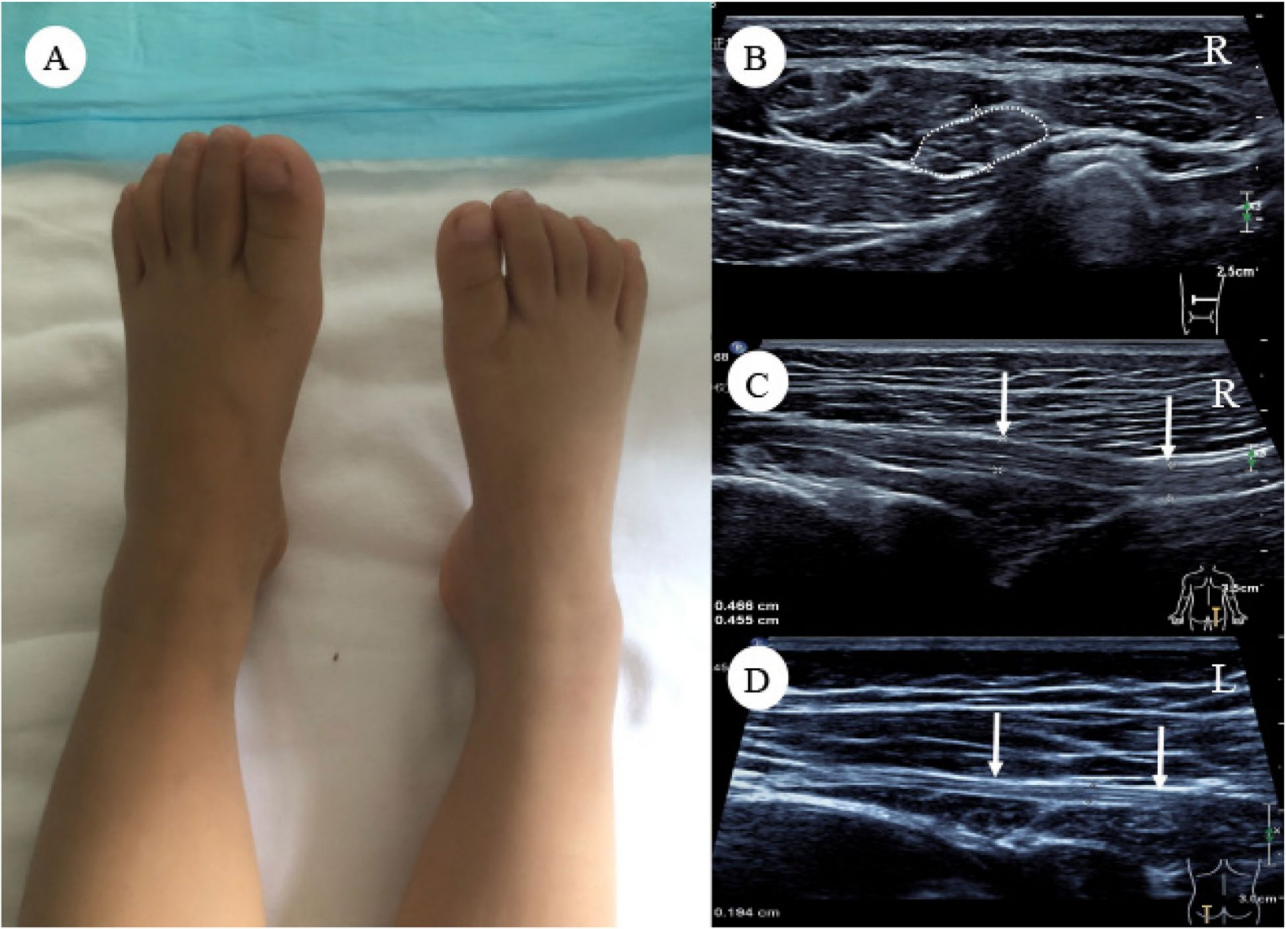
Images of patient #7. A 3-year-old girl with typical clinical manifestations of neuromuscular choristomas (NMC) of the sciatic nerve, including leg length discrepancy and calf muscles atrophy, and right foot shorter than the left foot (**A**). Ultrasound imaging demonstrated that the right sciatic nerve of the upper and middle thigh was hypoechoic and fusiformly enlarged. The cross-sectional area of the nerve was 0.68cm^2^ (**B**), and the diameter was 0.47 cm in the longitudinal section (**C**). Comparing with the other side, the size and echo intensity were normal in the left sciatic nerve (diameter 0.19 cm, cross-sectional area 0.23cm^2^) (**D**). Neither progression of NMC nor neuromuscular choristomas associated desmoid-type fibromatosis (NMC-DTF) was found during the 8-month follow-up

Clinical data are presented in Table 1 and Figs. 1, 2, 3 and 4. All the NMC-DTFs were hard and firm on palpation. Motor deficits occurred in 4 patients (57.1%); the main manifestations were foot drop and toe extension weakness (common peroneal nerve injury). Sensory deficits occurred in 1 patient (14.3%), and neuropathic pain was reported in 3 patients (42.9%). Six patients (85.7%) presented with limb undergrowth, 6 (85.7%) with muscular atrophy, and 5 (71.4%) with cavus foot deformity. None of the seven patients found bone dysplasia.

**Table 1:** Clinical and physical examination of NMC

Patient	Age/Sex	Location	Side	Motor deficits	Sensory deficits	Neuropathic pain	Limb undergrowth	Muscular atrophy	Cavus foot	Bone dysplasia
1	22/F	sciatic nerve	Left	N	N	Y	Y	Y	Y	N
2	2/M	sciatic nerve	Right	Y	Un	Un	Y	Y	Y	N
3	6/M	sciatic nerve	Left	Y	Y	Y	Y	Y	Y	N
4	10/F	brachial plexus	Left	N	N	Y	N	N	N	N
5	2/M	sciatic nerve	Right	Y	Un	Un	Y	Y	Y	N
6	4/M	sciatic nerve	Left	N	N	N	Y	Y	Y	N
7	3/F	sciatic nerve	Right	Y	N	N	Y	Y	N	N

*NMC* Neuromuscular choristomas, *Y*  Yes, *N* No, *Un* Unknown due to young ages

### Ultrasound imaging features

All ultrasound imaging features of NMC/NMC-DTF are summarized in Table 2. Based upon ultrasound findings, all visible NMC affected nerve segments presented with hypoechoic and fusiform enlargement (Figs. 1, 2, and 4). The echo intensity of the intraneural soft-tissue components was comparable to that of the surrounding skeletal muscle. One affected nerve segment was invisible due to massive NMC-DTF (Fig. 3). The structures of the perineurium and epineurium in the affected nerve were clear. The cross-section of the affected nerves still showed the same cribriform pattern as the normal nerve which was different from other peripheral nerve tumors (i.e., neurofibroma, schwannoma and perineurioma).

**Table 2:** Ultrasound imaging features of NMC/NMC-DTF

Patient	Age/Sex	Location	Morphology of involved nerve	Transformation to DTF	NMC-DTF size (cm)	NMC-DTF margin	NMC-DTF echo intensity and morphology	CDFI grade	Spatial relationship between NMC-DTF and involved nerves	Local recurrence
1	22/F	sciatic nerve	Hypoechoic, fusiform enlargement	Yes	12.6 × 6.3 × 3.4	Well-circumscribed	Hypoechoic solid lesion, irregular shape	1–2	NMC-DTF envelops the branches of the left sciatic nerve	No
2	2/M	sciatic nerve	Hypoechoic, fusiform enlargement, hyperechoic striated appearance in the nerve	Yes	13.6 × 4.4 × 3.8	Well-circumscribed	Solid lesion with heterogeneous internal echo, irregular shape	1–2	NMC-DTF envelops the proximal adjacent nerve	No
3	6/M	sciatic nerve	Hypoechoic, fusiform enlargement	Yes	24 × 17 × 12	Well-circumscribed	Hypoechoic solid lesion, irregular shape	1–2	NMC-DTF envelops the proximal adjacent nerve	Twice
4	10/F	brachial plexus	Nerve invisible due to massive NMC-DTF	Yes	5.5 × 5.5 × 3.3	Well-circumscribed	Heterogeneous hypoechoic solid lesion, irregular shape	1–2	Nerve structure is invisible due to massive NMC-DTF	No
5	2/M	sciatic nerve	Hypoechoic, fusiform enlargement	Yes	6.3 × 4.6 × 3.6	Well-circumscribed	Hypoechoic solid lesion, irregular shape	1–2	NMC-DTF envelops the proximal adjacent nerve	Once
6	4/M	sciatic nerve	Hypoechoic, fusiform enlargement	No	—	—	—	—	—	No
7	3/F	sciatic nerve	Hypoechoic, fusiform enlargement	No	—	—	—	—	—	No

*NMC *Neuromuscular choristomas, *NMC-DTF *Neuromuscular choristomas associated desmoid-type formation, *CDFI* Color Doppler blood flow imaging

Among 7 NMCs, five patients (71.4%) had NMC-DTFs at the site of the affected nerve (all previously underwent surgical resection or biopsy). No lesion grew to affect a new neighbouring nerve. All NMC-DTFs appeared as heterogeneous hypoechoic solid lesions adjacent to the nerve with irregular shapes, and they were all well circumscribed. The color Doppler blood flow was grade 1–2. Among the five NMC-DTFs, three enveloped the proximal adjacent sciatic nerve (Patients #2,3, and 5). One case enveloped the distal branches of involved sciatic nerve (Patient #1). One occurred in the brachial plexus, and the tumor was close to the vertebral body; the structure of the proximal nerve root was invisible on ultrasound (Patient #4). Postoperative pathology confirmed that the brachial plexus nerve root was running through the inside of the tumor which could not be distinguished from the tumor.

### Pathological findings

Five patients were diagnosed with NMC by nerve biopsy (1 patient) and/or previous surgical resection of NMC/NMC-DTFs (4 patients). Among the five available pathological results, all NMCs were composed of varying amounts of mature skeletal muscle fibers intercalated among the peripheral nerve fascicles. Based upon immunohistochemical staining, all NMCs showed scattered (myo) fibroblasts with aberrant nuclear localization of the β-catenin protein (Figs. 1, 2 and 3). One case was also positive for a CTNNB1 p. S45F mutation (Fig. 2; patient #3).

### Clinical course and follow-up

Clinical course and follow-up information are presented in Table 3. The mean clinical follow-up time after enrolment was 9.4 months with a range of 6–12 months. Progression of motor deficits and muscle atrophy was observed in 2 (28.6%) patients. One patient (14.3%) had progression of limb undergrowth. Tumor recurrence was detected in two patients (28.6%) at 3 and 8 months after the first surgical resection. One of the two patients underwent three tumor resections and a course of radiotherapy. The other patient underwent chemotherapy (twice) and high-intensity focused ultrasound knife (HIFU) treatment six rounds, which failed to control tumor progression and finally led to above-knee amputation (Fig. 2).

**Table 3:** Clinical course and follow-up

Patient	Age/Sex	Location	Surgery before	Transformation of Desmoid-type Formation(DTF)	Treatment process	Pathological finding	Follow-up	
							Tumor recurrence frequency	Current condition
1	22/F	sciatic nerve	Yes	Yes	Tumor resection (Once)	β-Catenin ( +)	0	No progression
2	2/M	sciatic nerve	Yes	Yes	Surgical biopsy of sciatic nerve	β-Catenin ( +)	0	Progression of motor deficit, limb undergrowth and calf muscles atrophy
3	6/M	sciatic nerve	Yes	Yes	Tumor resection (Once); chemotherapy (Two cycles); HIFU (Six rounds);	β-Catenin ( +) CTNNB1 p. S45F mutation	Once (At the site of NMC)	Continuous tumor progression lead to above-knee amputation
4	10/F	brachial plexus	Yes	Yes	Tumor and involved brachial plexus resection, nerve transplantation	β-Catenin ( +)	0	No progression after nerve transplantation
5	2/M	sciatic nerve	Yes	Yes	Tumor resection (Three times); radiotherapy (A course)	β-Catenin ( +)	Twice (At the site of NMC)	No progression after last treatment
6	4/M	sciatic nerve	No	No	None	—	0	No progression
7	3/F	sciatic nerve	No	No	None	—	0	No progression

*HIFU* High intensity focused ultrasound knife, *NMC* Neuromuscular choristomas

**Fig. 5:**
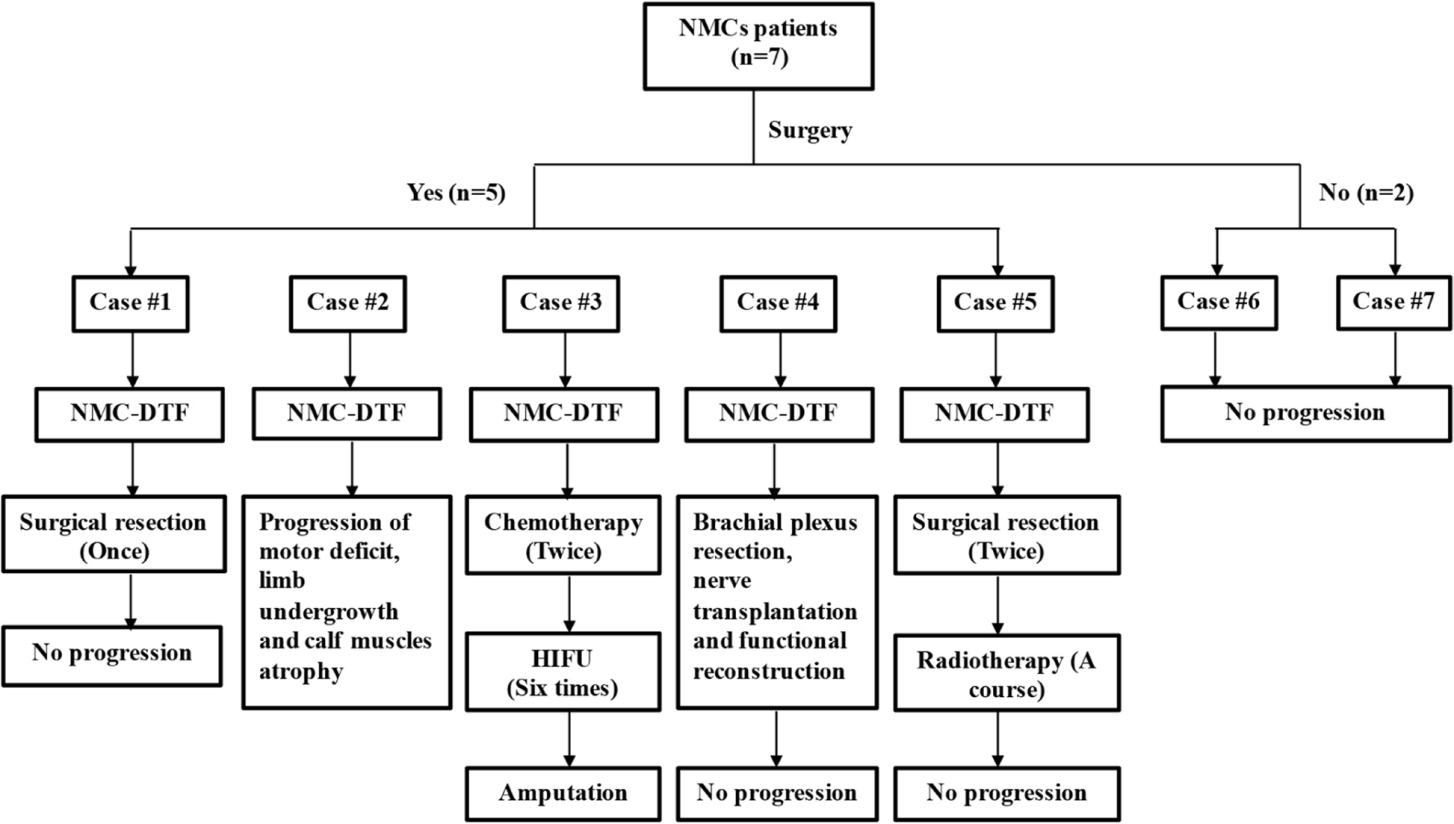
Flow chart of the treatment process and follow-up in seven patients

In the subset of the tumor surgery subset group, all 5 patients presented with progression of NMC-DTFs at the site of the NMCs. No fibromatosis was detected in the other two nonsurgical patients, and the disease was stable during the observation period (Fig. 5).

## Discussion

Neuromuscular choristoma (NMC) is a rare developmental lesion that typically involves major nerves or plexuses and most commonly affects the sciatic nerve and brachial plexus [1, 4–6, 13, 18, 19]. It is characterized by the biomorphic composition of heterotopic skeletal muscle fibers within the peripheral nerves. Unlike hamartomas, it contains mature muscle fibers in an aberrant location and may be best classified as a form of heterotopia [1]. Previous studies have reported that desmoid-type fibromatosis (DTF) at the site of the NMC may be incited by surgical resection/biopsy. Although it does not undergo metastatic transformation, NMC-DTF is locally aggressive and infiltrative, frequently encompassing neurovascular structures and leading to local recurrence after resection [16, 20].

Previous studies have reported that patients with NMC typically present in childhood with localized neuropathy or plexopathy and manifestations of chronic undergrowth in the affected nerve’s territory [16]. In our study, all the patients were under 25 years old, and most were under ten (85.7%). The detection rates of motor deficit, neuropathic pain, limb undergrowth, muscular atrophy, and cavus foot deformity were 71.4%, 42.9%, 71.4%, 85.7%, and 71.4%, respectively. The main manifestation of motor deficits was foot drop and toe extension weakness due to common peroneal nerve injury. All these symptoms were in the territory of the affected nerve.

Ultrasound examination could help to diagnose NMC without the need for a biopsy. Based upon our findings, all the visibly affected nerve segments presented with hypoechoic and fusiform enlargement. The structures of the perineurium and epineurium in the affected nerve were clear. Most importantly, the echo intensity of the intraneural soft-tissue elements was comparable to that of the surrounding skeletal muscle, and the cross-section of the affected nerves still showed a cribriform pattern similar to that of the normal nerve. This finding was consistent with the pathological structure of NMCs, since all NMCs were composed of various amounts of mature skeletal muscle fibers intercalated among the peripheral nerve fascicles. It may help us differentiate NMCs from other peripheral nerve tumors (i.e., neurofibroma, schwannoma and perineurioma). All NMC-DTFs were contiguous and showed a hypoechoic solid lesion adjacent to the enlarged nerve with an irregular shape. All tumors occurred at the site of the NMC-affected nerve and no lesion grew to affect any other anatomic site, which was consistent with previous reports [16].

In all five pathologically confirmed cases, we observed distinctive histologic features of NMC: varying amounts of mature skeletal muscle fibers inside the endoneurium, intercalated among the peripheral nerve fascicles, resulting in enlargement of the affected nerve segment [11]. Based upon a second review of immunohistochemical staining, all the stains showed aberrant nuclear localization of beta-catenin protein, which is an established indicator of activated CTNNB1 mutations (the gene that encodes the beta-catenin protein) [16, 21, 22] Previous studies have demonstrated that NMC and NMC-DTF both harbor identical CTNNB1 mutations, particularly CTNNB1 p. S45F, a specific CTNNB1 mutation that has been associated with more aggressive clinical behavior [21, 22]. In our study, the positive expression rate of beta-catenin protein was 100% in patients who received a confirmed diagnosis by surgical pathology. One case was also positive for a CTNNB1 p. S45F mutation and eventually led to an above-knee amputation. This may be one reason that all surgical resection patients presented with progression to NMC-DTFs, and half of them had local recurrence. This result implied that if a pathologic diagnosis of NMC was obtained, follow-up studies may be warranted to assess the development of aggressive fibromatosis. For patients with NMC, beta-catenin protein expression (CTNNB1 mutation, particularly CTNNB1 p. S45F) should be detected for a better outcome.

In our study, most of the patients underwent consultation for recurrent DTF and were found to have an occult underlying NMC. We agree with the opinion that the coexistence of NMC may be underrecognized in patients with extremity DTF [23]. Based on our course review and follow-up, all patients who underwent surgery presented with progression to NMC-DTFs at the site of the NMCs, while no fibromatosis was detected in nonsurgical patients. Previous studies have suggested that the potential for fibromatosis occurring after surgery might lead to a “no touch” approach when NMC is suspected, which means that the diagnosis should be based solely on clinical and imaging criteria [1, 23, 24]. Although the natural history and true incidence of NMC and NMC-DTF remain unknown, our study favors the belief that for NMC patients, diagnosis is possible prior to biopsy or resection based on unique and characteristic ultrasound findings with consistent clinical findings. On the other hand, peripheral nerves should be inspected carefully when examining imaging in all patients (especially for young patients) with extremity DTF to avoid missed diagnosis of NMC, as this may have different follow-up and treatment strategies [16]. Additionally, the advantage of ultrasound in young children is that there is no need for sedation and the patient can be monitored repeatedly without harmful radiation. Therefore, an accurate diagnosis based on ultrasound findings may dissuade the clinician from proceeding with an invasive procedure. And it may be necessary to closely follow up with the patient regarding the development of NMC-DTFs [25].

For patients with NMC-DTF, the correct treatment algorithm remains unknown. In the subset of our tumor surgery group, all 5 patients presented with progression of NMC-DTFs at the site of the NMCs. Previous studies supported that the mainstay of treatment for NMC-associated DTF could be nonsurgical management including locoregional chemotherapy, exclusive radiation, systemic chemotherapy, and/or targeted therapy [23, 26]. Further study may be needed to identify the best treatment of NMC-DTF.

Our study has several limitations. First, this study was small cohort due to the rarity of NMCs. More clinical studies and radiologic examinations may be performed to better recognize this disease. Second, pathological confirmation of NMC was obtained in 5 cases; two patients lacked a pathological diagnosis based on our current “no-touch” principle, and longer follow-up may be required to demonstrate the features and progression of NMCs. More features should be assessed by long-term prospective follow-up of a large sample size.

## Conclusions

In conclusion, NMC is a rare developmental disease that requires an accurate diagnosis in the appropriate clinical setting. Understanding the typical ultrasound features and clinically associated conditions would support early diagnosis and recommend an ideal treatment for NMC. Based on our experience and the literature, applying the “no touch” principle to the affected nerve might be an optimal suggestion for the occurrence of NMC in childhood.

## Data Availability

All the data needed to achieve the conclusion are contained within the paper. The raw data cannot be shared publicly due to ethical reason.

## Abbreviations

NMC: Neuromuscular choristomas
DTF: Desmoid-type fibromatosis
NMC-DTF: Neuromuscular choristomas associated desmoid-type fibromatosis
HIFU: High-intensity focused ultrasound knife
